# Improved Protoplast Production Protocol for Fungal Transformations Mediated by CRISPR/Cas9 in *Botrytis cinerea* Non-Sporulating Isolates

**DOI:** 10.3390/plants13131754

**Published:** 2024-06-25

**Authors:** Víctor Coca-Ruiz, Nuria Cabrera-Gómez, Isidro G. Collado, Josefina Aleu

**Affiliations:** 1 Departamento de Química Orgánica, Facultad de Ciencias, Universidad de Cádiz, 11510 Puerto Real, Cádiz, Spain; victor.coca@uca.es (V.C.-R.); nuriacabgom@gmail.com (N.C.-G.); 2 Instituto de Investigación en Biomoléculas (INBIO), Universidad de Cádiz, 11510 Puerto Real, Cádiz, Spain

**Keywords:** *Botrytis cinerea*, protoplast, CRISPR/Cas9, mycelium, transformation

## Abstract

*Botrytis cinerea* is a necrotrophic fungus that causes considerable economic losses in commercial crops. Fungi of the genus *Botrytis* exhibit great morphological and genetic variability, ranging from non-sporogenic and non-infective isolates to highly virulent sporogenic ones. There is growing interest in the different isolates in terms of their methodological applications aimed at gaining a deeper understanding of the biology of these fungal species for more efficient control of the infections they cause. This article describes an improvement in the protoplast production protocol from non-sporogenic isolates, resulting in viable protoplasts with regenerating capacity. The method improvements consist of a two-day incubation period with mycelium plugs and orbital shaking. Special mention is made of our preference for the VinoTaste Pro enzyme in the KC buffer as a replacement for Glucanex, as it enhances the efficacy of protoplast isolation in B459 and B371 isolates. The methodology described here has proven to be very useful for biotechnological applications such as genetic transformations mediated by the CRISPR/Cas9 tool.

## 1. Introduction

CRISPR/Cas9 technology has been increasingly used in the genetic engineering of fungi, particularly in the context of secondary metabolite production. This technology has been shown to accelerate the exploration and development of secondary metabolites from filamentous fungi [1]. The CRISPR/Cas9 system, composed of a Cas9 nuclease and a guide RNA molecule, has been successfully adapted to function in filamentous fungi such as *Aspergillus niger* and *Fusarium venenatum* and is considered one of the main drivers of progress in understanding various aspects of fungal biology [2,3]. This technology has also been used to edit the genomes of endophytic fungi and potentially increase their production of bioactive compounds [4]. The application of CRISPR/Cas9 in fungi has opened new paths, leading to the discovery and production of important secondary metabolites by overcoming previous limitations in genetic engineering in fungi [5]. Examples of fungi that have been genetically modified using CRISPR/Cas9 for this purpose include *Talaromyces atroroseus*, *Aspergillus* species, *Trichoderma reesei*, and *Ustilago maydis* [1,6,7].

Both endonuclease and sgRNA must coexist within the nucleus of the specific organism to spark Cas9-mediated genome editing. A range of strategies, including the following commonly used methods, have been used to introduce these components into filamentous fungi and oomycetes: polyethylene glycol (PEG)-mediated transformation, the most frequently used in several genera of fungi such as *Alternaria*, *Penicillium*, *Fusarium* and others [2,8,9], PEG-*Agrobacterium tumefaciens*-mediated transformation [10,11,12,13], PEG-mediated spore transformation [14], *Agrobacterium tumefaciens*-mediated transformation [15,16,17], electroporation [18,19] and electroporation with biolistic transformation [20,21]. All these methods have been used to edit the genomes of filamentous fungi and have proven valuable in enhancing the study of fungal biology. These data were previously reported by Schuster and Kahmann in 2019 [22]. 

The main delivery methods for Cas9 and sgRNA involve: (i) integrating DNA encoding of both Cas9 and sgRNA into the genome or providing them as components within plasmids, (ii) introducing DNA-carrying Cas9 followed by the introduction of sgRNAs transcribed in vitro, and (iii) delivering a preformed CRISPR/Cas9/sgRNA ribonucleoprotein (RNP) complex through transformation [22]. After transformation, the Cas9 gene can either be integrated stably into the genome at random sites or targeted to specific sites [9,20]. In both cases, a selection marker is co-transformed [23]. Alternatively, the protein Cas9 can be transiently transformed as an integral part of a plasmid that lacks both origins of replication and homology to the host genome. This type of plasmid is introduced into cells without any specific selection but is delivered together with donor DNA carrying a selectable marker [10,18,22].

Protoplast-mediated transformation (PMT) is widely used to genetically manipulate filamentous fungi. This involves preparing protoplasts—fungal cells with their cell walls removed—and transforming them with foreign DNA, often facilitated by enzyme treatment. PEG-mediated protoplast transformation is a mature and effective approach to genetic manipulation, enabling CRISPR/Cas9 genome editing [24,25]. Protoplast-based genetic transformation systems prove crucial in studying gene function and metabolic processes in fungi [26]. Optimization is required for different fungal species due to the specific characteristics of cell wall structures [27,28]. Protoplasts, utilized for CRISPR/Cas9 mutagenesis in plants, are particularly effective, allowing direct gene editing in the T0 generation [29]. This method has been successfully applied in organisms such as *B. cinerea*, showcasing its versatility in genetic editing across different species [30,31].

The *Botrytis* genus comprises a diverse group of pathogenic fungal species distributed worldwide [32,33]. Characterized by their gray mycelium and saprotrophic behavior, *Botrytis* species act as necrotrophic pathogens, causing substantial losses in various economically significant horticultural and floral crops [34,35].

*B. cinerea* protoplasts have been successfully produced from young mycelium of freshly germinated spores [36,37,38], and various transformation methods have been developed [39,40,41]. These methods have facilitated functional genetic analysis of *B. cinerea* and have contributed to wide-ranging molecular research on this pathogen [42]. In that way, the first protocol described for protoplast isolation from *B. cinerea* to transform the fungus was reported by Hamada et al. in 1994 [40]. This protocol was based on the incubation of freshly germinated mycelium (23 °C during 2 h in a rotary shaker with 80 rpm) with a lytic enzyme (Novozymes) capable of degrading the cell wall and the subsequent release of protoplasts. Different protocols for protoplast isolation have been reported based on modifications of Hamada et al. in the type of lytic enzyme used, time and type of agitation of the lytic reaction. Zhang et al. in 2014 or Reis et al. in 2005 used 0.5% Glucanex as the lytic solution and incubated the protoplast mixture at 25 °C and 100 rpm for 2 h to produce protoplasts [43,44]. Braun and Heisler in 1990 performed *B. cinerea* protoplast isolation from not fresh germinated spores (spores after 48 h incubation) incubating in an enzyme mix based on 0.4% glucuronidase, 0.4% gellulase RIO, 0.2% Driselase and 0.6 M mannitol [37]. The addition of zymolase (lyticase) to the enzyme solution, as published by Shirane and Hatta (1986), reduced the incubation time by only 1 h but did not increase the production [36,37]. The use of Cellulase RIO, Cellulase RS or Cellulase TC during lysis also gave no difference in production [37]. In addition, the use of Macerozyme and Pectinase was also ineffective, and protoplasts could be isolated without the use of the Driselase enzyme [37]. On the other hand, under the condition of spores after 48 h of incubation (no fresh germinated spores), the isolation method using Novozym 234, as described by Harling et al. (1988), was tested [45]. The protoplasting reaction was reduced in time 2 h but resulted in the obtention of unstable protoplasts [37]. However, protoplast isolation from spores germinated after 48 h did not give as good results as isolation from freshly germinated spores (spores with an incubation time of 16–20 h) [37,43,44]. Finally, the latest version of protoplast isolation applied to CRISPR/Cas9 was performed by Leisen et al. in 2020 by incubating freshly germinated spores with 1% Glucanex and 0.1% Yatalase and incubating them on a 3D rotary shaker at 60 rpm for 60–90 min at 28 °C [30].

On the other hand, some wild *B. cinerea* isolates from vineyards are characterized by their inability to sporulate [46]. This has prompted interest in the potential application of biotechnology to the study of these isolates, with the aim of improving our understanding of the biology of this phytopathogen. For this reason, this article reports a novel update in the protocol used to protoplast isolation from species unable to sporulate with a view to performing transformations with CRISPR/Cas9 on non-sporulating isolates of *B. cinerea*.

## 2. Results

### 2.1. Optimization of the Protocol for Protoplasts Isolation from Non-Sporulating B. cinerea Isolates

**Figure 1:**
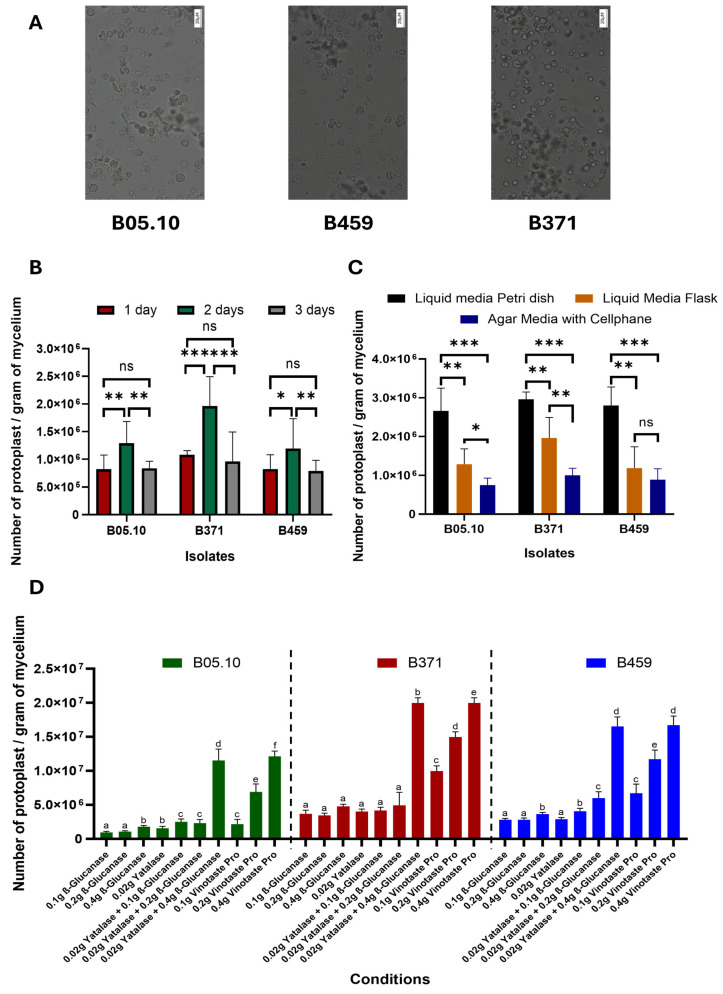
Optimization of protoplast production in *B. cinerea* non-sporulating isolates. (**A**) Image of protoplasts from the isolates B05.10, B459 and B371 (scale: 20 µM). Representation of the number of protoplasts per gram of mycelium of the three isolates B05.10, B459 and B371 when varying the time (**B**), culture method (**C**) and type of enzyme used for protoplast isolation (**D**). The results for (**B**–**D**) are the average of five independent biological replicates for each isolate. In (**B**,**C**), ns means *p* > 0.05, * means *p* < 0.05, ** means *p* < 0.01 and *** means *p* < 0.001. Different letters in (**D**) for each strain on the top of the bars represent significant differences (*p* < 0.05) between conditions.

Three parameters were optimized for protoplast isolation from non-sporulating *B. cinerea* isolates. Three *B. cinerea* isolates were used in this study: B05.10 as the control-sporulating strain (reference isolate from which protoplasts are routinely isolated) and isolates B371 and B459 as non-sporulating strains. We describe the first time protoplasts were isolated from isolate B459 (Figure 1), which does not show significant differences in terms of size and morphology compared to isolates B371 and B05.10 (Figure 1).

However, there were significant differences with respect to the time the protoplasts were left to incubate. Of the times tested (one, two and three days), two days are optimal to isolate the highest number of protoplasts for the three isolates tested (Figure 1B). Likewise, isolate B371 is the one exhibiting the greatest differences between times for protoplast isolation, while the significance of these differences for isolate B459 is not as high as in the case of B371. Isolate B05.10 behaved similarly to B459 (Figure 1B).

Regarding culture methods used for protoplast isolation, liquid medium in a Petri dish with orbital shaking at 40 rpm clearly resulted in higher protoplast yield than liquid medium in a 500 mL flask with orbital shaking at 180 rpm. Lastly, the plate culture method using a solid medium covered with cellophane was the least effective in protoplast isolation (Figure 1C). This held true for all three isolates except for isolate B459, where the solid medium method covered with cellophane and the liquid medium in 500 mL flask and orbital shaking at 180 rpm do not show significant differences between them (Figure 1C).

Lastly, with respect to the combination of enzymes used, all three isolates behaved similarly. The enzymes tested individually were not effective in protoplast isolation, except for VinoTaste Pro, which produced the highest yield when it was used at 0.4 g. The combination of β-Glucanase and Yatalase, with a high concentration of β-Glucanase, was just as effective in protoplast isolation as the individual enzyme VinoTaste Pro at the highest concentration tested (Figure 1D). Notably, the VinoTaste Pro enzyme used on its own at low concentrations led to comparable protoplast isolation as the combined use of β-Glucanase and Yatalase, making it the best enzyme tested in terms of the number of protoplasts isolated (Figure 1D). However, protoplast isolation using VinoTaste Pro at low concentrations is not as effective for B05.10 as for the rest of the isolates (Figure 1D).

### 2.2. Optimized Protocol for Protoplast Regeneration

**Figure 2:**
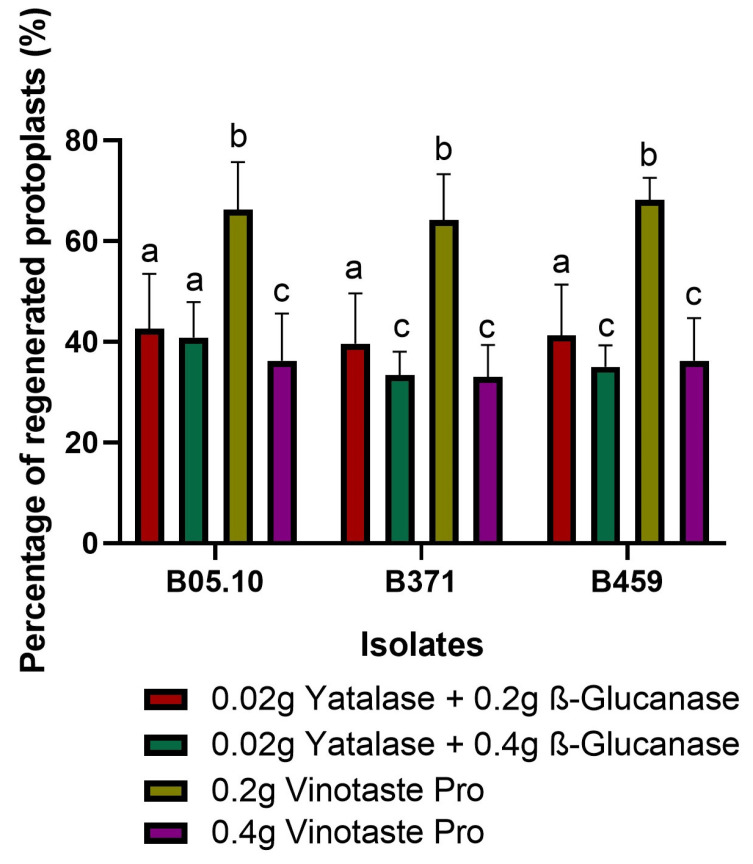
Protoplast regeneration. Percentage of regenerated protoplasts for isolates B05.10, B371, and B459 using the enzyme combination producing the maximum number of protoplasts. The graph shows the average of five independent biological replicates for each isolate. Different letters among the bars represent significant differences *p* < 0.05 between different conditions (enzymes) for each isolate.

As in the final optimization VinoTaste Pro enzyme and the combination of β-Glucanase and Yatalase gave the highest number of protoplasts (Figure 1D), and the two best concentrations of these two types of protoplasting enzymes were used to observe protoplast regeneration. We determined 0.2 g of VinoTaste Pro to be the best option to isolate intact protoplasts with higher regeneration capacity (Figure 2). Another interesting fact is that the 0.4 g of VinoTaste Pro, described as the condition under which the highest number of protoplasts were isolated (Figure 1D), does not generate intact protoplasts, the regeneration capacity being similar to that resulting from the combination of β-Glucanase and Yatalase enzymes (Figure 2). This suggests that the use of the VinoTaste Pro enzyme at high concentrations can be overly aggressive in protoplast isolation for all three isolates.

### 2.3. Protoplast Suitability for Transformation by CRISPR/Cas9

The integration of protoplasts with the CRISPR/Cas9 transformation technique serves as a powerful tool for genetic editing in plants and microorganisms. This technique enables the precise introduction of genetic changes such as insertions, deletions, or specific modifications in DNA, allowing for the controlled modification of key characteristics in living organisms. The isolated protoplasts were transformed to test their utility and assess three key aspects: random insertion of foreign DNA, ability to repair damage with foreign DNA, and CRISPR/Cas9-mediated targeted integration.

(1) Random Insertion of Foreign DNA: Protoplasts were transformed to observe whether foreign DNA could be randomly inserted into their genomes. Using the pNDH-OGG plasmid and PCR testing, the integration of the hygromycin resistance cassette (*hph*) at random sites in the genome was analyzed. This helps us understand the efficiency and likelihood of foreign DNA integration at various genomic locations (Table 1 and Supplementary Materials Figures S1A and S2A).

(2) Ability to Repair Damage with Foreign DNA: Protoplasts were tested to evaluate their ability to repair DNA damage using foreign DNA as a template. This contributes to our understanding of DNA repair mechanisms and the efficiency of using exogenous DNA for repair processes (Table 1 and Supplementary Materials Figures S1A and S2A).

(3) CRISPR/Cas9-mediated Targeted Integration: Utilizing CRISPR/Cas9, targeted integration of specific DNA sequences was attempted in protoplasts. This involves selective modifications or insertions at precise genomic locations, showcasing the accuracy and efficacy of CRISPR/Cas9 in effectuating genetic alterations (Table 1 and Supplementary Materials Figures S1B and S2B).

These experiments provide insights into the behavior and functionality of protoplasts regarding their suitability for genetic manipulation. Understanding these aspects helps assess the feasibility and potential of protoplast transformation for various genetic engineering applications, including the integration of foreign DNA into the genome, DNA repair studies and targeted gene editing.

The random integration of the hygromycin cassette (*hph*) was identified through PCR analysis using primer pairs CKhphpndhFW/CKhphpndhRv (141 bp) across three isolates (Supplementary Materials Figure S2A). Subsequent diagnostic PCR confirmed the occurrence of homologous integration events and the absence of *BcniaD* alleles in isolates B05.10, B459, and B371 (Table 1 and Supplementary Materials Figure S2B). The absence of *BcniaD* alleles in the mutants was verified using primers CKniaDhph/CKniaDout (1174 bp), while the presence of *BcniaD* alleles in the mutants was confirmed using primers CKniaDin/CKniaDout (423 bp) (Table 1 and Supplementary Materials Figure S2B). For both random integration and the capability to repair damage with exogenous DNA, the positive control involved amplification with primers CKhphpndhFW/CKhphpndhRv using the pNDH-OGG plasmid, while the negative control was amplification with the same primers on the wild-type isolate B05.10 (Supplementary Materials Figure S2A). Conversely, for targeted integration mediated by the CRISPR/Cas9 tool, the positive control entailed amplification in different wild-type isolates using primers CKniaDin/CKniaDout, while the negative control involved amplification with primers CKniaDhph/CKniaDout in the different wild-type isolates (Supplementary Materials Figure S2B).

The transformation success rate of targeted integration mediated by CRISPR/Cas9 was above 90% for all three isolates, with approximately 13–19% of the colonies exhibiting homokaryotic characteristics (Table 1). However, the success rate for random integration and repair of damage using foreign DNA did not exceed 20% for the three isolates (Table 1). The negative controls used in the transformation result in a slightly lower number of false positives than when transforming with the pNDH-OGG plasmid alone, indicating the effectiveness in the transformation mechanism of *B. cinerea*, in particular that mediated by CRISPR/Cas9 (Table 1).

These data suggest that CRISPR/Cas9-mediated targeted integration is highly efficient, resulting in a significant proportion of transformed colonies displaying the desired genetic alterations. In contrast, random integration of foreign DNA and repair with a foreign DNA template appears to have lower efficiency rates, suggesting potential limitations in these processes or the need for optimization to enhance their success rates.

Understanding these divergent degrees of efficiency across different genetic manipulation methods is crucial for optimizing protocols and further improving the success rates of random integration and repair mechanisms using foreign DNA. It highlights the robustness of CRISPR/Cas9 for precise genetic modifications compared to random integration or DNA repair processes.

## 3. Discussion

Protoplast isolation is a technique used in biology to isolate and study the contents of a cell by removing its cell wall leaving behind the plasma membrane and its contents. This technique is particularly valuable in cell biology, genetics, and biotechnology research for applications such as transformation studies, genome engineering and plant breeding [47]. Optimizing the protocols for protoplast isolation envisaging high yields is pivotal for efficient genetic manipulation in various fungal species, enabling advancements in fundamental research and biotechnological applications [48,49]. One such extensively studied filamentous fungus is *Aspergillus nidulans*, which serves as a model organism for understanding fungal biology and as a platform for developing novel biotechnological processes [50]. Protoplasts are essential intermediates for genetic transformation, allowing for the introduction of exogenous DNA and subsequent targeted gene editing [24].

Enzymatic digestion plays a central role in protoplast isolation, involving the selective degradation of the fungal cell wall while preserving cell viability [51]. Fine-tuning enzyme concentrations, incubation times, and temperature conditions are essential parameters for optimizing the efficiency of cell wall digestion [52]. For instance, a combination of cell wall-degrading enzymes such as Driselase, Lysing Enzymes, or Cellulase is commonly used [53]. Additionally, optimizing the duration of enzymatic treatment and incubation temperature ensures optimal protoplast yield and viability [54].

Moreover, the inclusion of suitable osmotic stabilizers in the isolation buffer is crucial for maintaining protoplast integrity and viability [55]. Osmotic stabilizers such as sorbitol or sucrose help counteract the osmotic shock experienced by protoplasts upon cell wall removal, thereby preventing cellular rupture and improving survival rates [56]. Optimizing the concentration of osmotic stabilizers is essential, as excessive concentrations may lead to osmotic stress, while inadequate concentrations may result in protoplast swelling or lysis [57]. Furthermore, purification steps are integral to isolating pure and viable protoplast populations. Filtration and centrifugation are commonly employed techniques for removing undigested cell debris and mycelial fragments, thus enhancing the purity and viability of the protoplast suspension. Careful attention to purification methods minimizes contamination and ensures the success of subsequent transformation experiments [49].

Several studies have contributed valuable insights and methodologies for optimizing protoplast isolation protocols in *Aspergillus* species. Cui (2022) demonstrated the importance of optimizing enzyme concentrations and osmotic stabilizers for efficient protoplast isolation in *Aspergillus niger* [58]. Similarly, Zhao et al. (2019) provided detailed protocols for protoplast isolation and transformation in *Aspergillus fumigatus* [59], highlighting the significance of purification steps in high-quality protoplast isolation. Additionally, Yu et al. (2015) elucidated the role of osmotic stabilizers in maintaining protoplast viability during the isolation process in *Ustilago esculenta* [56], offering valuable insights for protocol optimization.

In this work, three parameters were optimized for optimal protoplast isolation. First, the optimal time for protoplast isolation from mycelial plugs was determined to be two days (Figure 1B). An incubation time of one day resulting in a younger mycelium was not as effective as an incubation time of three days, which allowed the mycelium to mature but, contrary to what would be expected, isolation success decreased drastically. This two-day incubation period differs from the classical protoplast isolation protocols in *B. cinerea* B05.10 (from freshly germinated spores), where protoplasts are left to incubate for only one day [36,37,38]. It is noteworthy that conidia growth is much faster than mycelial plug growth in liquid medium since each conidium will give rise to a new fungus.

Second, different culture methods were evaluated, the liquid culture in a Petri dish and at an agitation of 40 rpm proving optimal, as opposed to the classical method of protoplasts isolation from freshly germinated spores where 100 mL of liquid medium is used in 500 mL flasks with agitation of 180 rpm. This method reported for protoplast isolation is an adaptation of the classical method described by Celedonio González et al. to obtain genomic DNA from *B. cinerea* B05.10 [60]. The solid plate culture method covered with cellophane was not effective.

Third, different types of enzymes were evaluated to determine the optimal candidate to replace the classic Glucanex used in *B. cinerea* transformations, which, for commercial reasons, is no longer available [40]. VinoTaste Pro was the enzyme with which the highest number of protoplasts was isolated. In addition, optimization tests were performed regarding the concentrations used to extract protoplasts since, as can be seen in Figure 2, the highest concentration applied was not ideal for maintaining the viability or regenerative capacity of the protoplasts. An ideal concentration of 0.2 g of enzyme in 20 mL of KC buffer for 2 g of mycelium was determined. This coincides with the classic concentration of the now-discontinued Glucanex for protoplast isolation. Higher concentrations of this enzyme diminish protoplast viability due to its aggressiveness.

Regarding other protoplast isolation methods described for *B. cinerea*, they all begin with germinated conidia and not mycelium. This work presents a novel method for isolating protoplasts (useful for transformation) from propagated mycelium (and not mycelium from germinated spores). The highest number of functional protoplasts isolated in this study was 2 × 10^7^ protoplasts per gram of mycelium for the condition of conducting the protoplast reaction with 0.2 g of VinoTaste Pro (Figure 2). This number of isolated protoplasts is higher than the protoplast production from other described protocols. In the case of Braun and Heisler, they used mycelium from 48 h germinated spores to extract the protoplasts, incubating the mycelium in a protoplast lytic solution composed of glucuronidase 0.4%, Cellulase RIO 0.4%, Driselase 0.2% and mannitol 0.6 M at 25 °C for 4–6 h, resulting in an amount between 2.6 × 10^6^ and 5.8 × 10^6^ protoplasts per gram of mycelium [37]. Our method reduces the protoplasting time by 4–6 h, resulting in 10 times more protoplasts by the utilization of the VinoTaste Pro enzyme (Figure 1D) when it was used at 0.4 g. On the other hand, with respect to a more optimized method of protoplast isolation for CRISPR/Cas9 transformation, as reported by Leisen et al. (2020), the number of protoplasts isolated from freshly germinated spores and incubation with an enzyme mixture composed of 1% Glucanex and 0.1% of Yatalase during 60–90 min at 28 °C, was approximately 2.5 × 10^7^ protoplasts per gram of mycelium [30]. This production was identical to that reported by Zhang et al. in 2014, where freshly germinated spores were incubated in only the lytic enzyme Glucanex at 25 °C and 100 rpm agitation for 2 h [44]. The production yields isolated by Leisen et al. in 2020 and Zhang et al. in 2014 [30,44] are very similar to the number of protoplasts isolated by the proposed method using the VinoTaste Pro enzyme (Figure 1D).

VinoTaste Pro is also used for the rapid clarification of red and white wines. It significantly reduces maturation time by nearly 20%. It also stabilizes color, enhances aroma and increases the roundness of wines [61]. Purified to be free of FCE activity, VinoTaste Pro eliminates the risk of off-flavors [62]. Consequently, the cost of obtention of this enzyme is very low, as it is used in industrial processes of wine production [62]. The alternative enzymes, such as β-Glucanase and Yatalase, present a significant quantity-price limitation, limiting the number of transformations that can be carried out [61]. The optimal solution for protoplast isolation is the use of VinoTaste Pro, which can be used without the need to combine it with other enzymes. This is followed by the combination of β-Glucanase and Yatalase. VineTaste Pro represents an economical and efficient alternative to the traditional enzyme Glucanex, which has been discontinued.

Isolated protoplasts can be used for various purposes. As the study shows, these isolated protoplasts were good for the CRISPR/Cas9 tool-mediated transformation of *B. cinerea*. The transformation was a success in over 90% of the scanned colonies, and between 10% and 20% of homokaryotic colonies were obtained from the total number of colonies analyzed. Comparing this transformation method with other methods carried out in *B. cinerea* reveals that the sclerotium-mediated transformation and direct hyphal transformation methods achieved transformation efficiencies of 46% and 39%, respectively, compared to 90% observed in CRISPR/Cas9-mediated protoplast transformation method [39]. This demonstrates that CRISPR/Cas9-mediated protoplast transformation is the most effective method to date for generating transformants in *B. cinerea*.

Hence, this is an effective method for the transformation of isolates incapable of sporulation and paves the way for the study of cellular processes, genetic manipulation, fusion experiments, and biotechnological applications among non-sporulating isolates, particularly B349 and B371, which are of great interest due to their inability to sporulate and infect and to understand the biology of this fungal species [46] with the final objective of obtaining an effective control method with direct application in agriculture.

This technique has broad implications in biotechnology and research, enabling the study and manipulation of cells that would otherwise be difficult to access or modify due to the cell wall.

## 4. Materials and Methods

### 4.1. Microorganisms and Growth Conditions

The B459 and B371 isolates of *B. cinerea* referenced in this study are integral components of the field isolate repository curated by CIALE (University of Salamanca, Spain). These isolates were collected and purified as described by Acosta Morel et al. [46]. *B. cinerea* B05.10 was kindly provided by Prof. Tudzynski [63]. MEA-modified (2% malt extract, 2% glucose, 0.1% peptone and 2% agar, pH 6.5) plates were used for the culture of *B. cinerea* isolates, which were incubated either under conditions of continuous darkness or continuous light at a temperature of 22 °C.

Protoplast optimization entailed the incubation of mycelium agar plugs in HA medium (4 g glucose, 4 g yeast extract and 10 g malt extract in 1 L of H_2_O, pH 5.5). For the generation of null mutants, SH-Agar medium (41.08 g sucrose, 1 mL Tris-HCl pH 6.5 1 M, 23 mg (NH_4_)H_2_PO_4_ and 1.8 g Bacto-Agar (ref.11758223, Becton Dickinson, Sparks, MD, USA) in 200 mL of H_2_O) supplemented with 17.5 μg/mL of hygromycin B (ref.10687010, Invitrogen, Waltham, MA, USA) served as the selection medium.

### 4.2. Time Optimization for Protoplast Obtention

To evaluate the ideal amount of time needed for isolating protoplasts, five mycelium plugs of each isolate were deposited in 100 mL of HA medium in 500 mL flasks (a procedure commonly used to extract protoplasts from freshly germinated spores of the isolate B05.10). They were incubated at 180 rpm for one, two and three days. Protoplasts were isolated according to the protoplast collection protocol described below in Section 4.5 using a unique enzyme (0.2 g β-Glucanase from *Trichoderma longibrachiatum* (Sigma-Aldrich, Burlington, MA, USA) and 0.02 g Yatalase (ref.T017, Takara, Kyoto, Japan)) for the three isolates (classical enzyme combination used to get the protoplast in the isolated B05.10 from freshly germinated spores, except for the substitution of Glucanex (ref.L1412, Sigma-Aldrich, Burlington, MA, USA) for β-Glucanase from *Trichoderma longibrachiatum* (ref.G4423, Sigma-Aldrich, Burlington, MA, USA) due to Glucanex discontinuation). The results were presented as the mean of the five independent biological replicates.

### 4.3. Type of Culture Optimization for Protoplast Isolation

Five mycelium plugs from each isolate were incubated in three different culture forms to evaluate the best culture form for protoplasts isolation:

- Five mycelium plugs over 100 mL of HA medium in 500 mL flasks and 180 rpm orbital shaking (classical method for protoplasts isolation from the isolate B05.10 from freshly germinated spores).
- Five mycelium plugs over 20 mL of HA medium in Petri dishes and 40 rpm orbital shaking.
- Five mycelium plugs on 20 mL of HA medium supplemented with 2% agar and covered with a layer of cellophane.

The three culture methods were incubated for two days, the optimal period of time for isolating protoplasts (see Section 2.1). The protoplast isolation method described in Section 4.5 using a unique enzyme (either 0.2 g of β-Glucanase from *Trichoderma longibrachiatum* or 0.02 g of Yatalase for the three isolates) was used to determine the best method of culture for protoplasts isolation. Results are presented as the mean value of the five independent biological replicates.

### 4.4. Enzyme Optimization for Protoplast Isolation

To test for the best enzyme for isolating protoplasts, five mycelium plugs from each isolate were incubated for two days in 20 mL of medium in Petri dishes at 40 rpm of orbital shaking (see Section 2.1 for the best period of time and best culture method for protoplast isolation). Protoplasts were isolated following the protocol described in Section 4.5. In this case, different enzyme concentrations were evaluated to determine the best concentration for protoplasts isolation from each of the isolates. Enzymes were tested individually at the following concentrations: 0.02 g of Yatalase, 0.1 g, 0.2 g and 0.4 g of β-Glucanase from *Trichoderma longibrachiatum* with and without the addition of 0.02 g of Yatalase, and 0.1 g, 0.2 g and 0.4 g VinoTaste^®^ Pro (ref.250g, Novozymes Spain S.A., Madrid, Spain). Results are presented as the mean value of the five independent biological replicates.

### 4.5. Protoplast Preparation

Mycelium was collected by filtration and washed three times with buffer KC (450 mL KCl 0.6 M and 50 mL NaPi 1 M pH 5.8 consisting of 15.8 mL Na_2_HPO_4_, 1 M and 184.2 mL NaH_2_PO_4_ 1 M pH 5.8). After each wash, the mycelium suspension was centrifuged for 5 min at 1000× *g*, discarding the supernatant. Enzymes (Yatalasa, β-Glucanase or VinoTaste Pro) were dissolved in the appropriate osmotic stabilizer (KC buffer) and sterilized by filtration through 40 µm-pore membranes (ref.SLGPR33RS, Merck Millipore Ltd., Tullagreen, Ireland). For enzymatic treatments, mycelium was suspended in the enzyme solution (10 mL for each fresh weight gram of mycelium) and incubated at 28 °C in darkness in a 500 mL flask under 85 rpm orbital shaking conditions. After 1 h of incubation, the protoplast suspension was filtered through 40 µM-pore membranes into a 50 mL falcon tube containing 10 mL ice-cold TMS buffer (91 g sorbitol (ref.S1876, Sigma-Aldrich, Burlington, MA, USA), 1045 mg MOPS (3-(N-morpholino) propanesulfonic acid) (ref.69947, Sigma-Aldrich, Burlington, MA, USA), pH 6.3). It was washed once with TMS (centrifuged for 5 min at 4 °C 1500× *g*) and filled to a volume of 5 mL of TMS. From that 5 mL, a 10 µL aliquot was used to count protoplasts in a hemocytometer. The remaining volume was centrifuged and resuspended in the desired volume of TMSC (18.2 g sorbitol, 209 mg MOPS and 588 mg CaCl_2_, pH 6.3).

### 4.6. Protoplast Regeneration

A protoplast suspension (in TMSC) with a concentration of 200 protoplasts per mL was prepared for each isolate to test for protoplast integrity. Protoplasts for this experiment were acquired after two days of incubation in a Petri dish with HA medium at 40 rpm. The enzymes and respective amounts used to extract the protoplasts evaluated in this experiment were 0.2 g and 0.4 g of β-Glucanase from *Trichoderma longibrachiatum* with 0.02 g of Yatalase and 0.2 g and 0.4 g of VinoTaste^®^ Pro (conditions presenting the highest yields of protoplast isolation). 100 µL of this protoplast suspension was added to 50 mL of SH-agar medium (at 40 °C) and distributed in four Petri dishes. After three days of incubation, the number of colonies that grew out of the total number was determined. The results are expressed as the mean percentage of protoplasts regenerated within each isolate under each condition, derived from five distinct and independent replicates.

### 4.7. Evaluation of Protoplast Usefulness by CRISPR/Cas9 Transformation

To evaluate the usefulness of the isolated protoplasts, we proceeded to transform them in two different ways. The plasmid pNDH-OGG was used as foreign DNA for both of these [64].

First, the random integration of foreign DNA into the genome of the three isolates was evaluated. For this purpose, only the plasmid pNDH-OGG was transformed into the protoplasts, causing random integration in the genome of the three strains (Supplementary Materials Figure S1A).

Second, the ability to regenerate genome damage by taking foreign DNA was evaluated. For this purpose, the pNDH-OGG plasmid was co-transformed together with the EnGen^®^ Spy Cas9 NLS (ref.M0646T, New England Biolabs, Ipswich, MA, USA) enzyme without sgRNA causing nonspecific cuts in the genome (Supplementary Materials Figure S1A).

Lastly, the plasmid pNDH-OGG was used as donor DNA to achieve double-strand break (DSB) direct recombination. This plasmid contains the GFP under the control of the OliC promoter from *Aspergillus nidulans* and the β-Glucanase terminator from *B. cinerea* joined to the hygromycin resistance cassette [65] flanked by the *B. cinerea* niaD flanks for proper integration at that locus (Supplementary Materials Figure S1A,B). For targeted Cas9-mediated DSB, sgRNA were designed and generated as described by Leisen et al. [30] and purified using the RNA Clean & Concentrator-100 kit (ref. R1019, Zymo Research Orange, Tustin, CA, USA). The oligos sgRNAConstantOligo [66], sgRNA1niaD, sgRNA2niaD and sgRNA3niaD [67] were used for this purpose (Supplementary Materials Table S1). A negative control without the addition of the plasmid pNDH-OGG was used as a control for the three transformations.

*B. cinerea* isolate transformation was performed as described [30] with some modifications corresponding to protoplast isolation. Five mycelium plugs of each isolate (instead of freshly germinated spores), incubated for 2 days in a Petri dish at 85 rpm shaking, and VinoTaste Pro enzyme were used to isolate the protoplast from the mycelium. Assembly of the ribonucleotide-protein (RNP) complex was performed as described [30] by adding 2 µg of each sgRNAs to 6 µg of Cas9 protein in cleavage buffer (20 mM HEPES (ref.H0887, Sigma-Aldrich, Burlington, United States), pH 7.5, 100 mM KCl, 5% glycerol, 1 mM Dithiothreitol (DTT) (ref. D0632, Sigma-Aldrich, Burlington, MA, USA), 0.5 mM Ethylenediaminetetraacetic acid (EDTA) (ref.E9884, Sigma-Aldrich, Burlington, MA, USA), pH 8 and 2 mM MgCl_2_). After RNP treatment, protoplasts were plated in SH medium containing 17.5 µg/mL hygromycin B and incubated in light conditions at 25 °C.

After four days of incubation, the fungal colonies that appeared were characterized using Phire Plant Direct PCR Kit (ref. F-130WH, Thermofisher Scientific, Waltham, MA, USA) and two primers to check the random integration of the *hph* gene into the genome of the three isolates: CKhphpndhFw and CKhphpndhRv. (Supplementary Materials Table S1). As a positive control, the pNDH-OGG plasmid was used as template DNA, while the genomic DNA from the B05.10 wild-type strain was used as a negative control (Supplementary Materials Figure S1A). For CRISPR/Cas9-mediated transformation, primer CKniaDout was used outside the niaD flank and primer CKniaDin in the wt locus to check for wt copies (Supplementary Materials Table S1 and Supplementary Materials Figure S1B). Primer CKniaDout, outside the niaD locus, and primer CKniaDhph in the hph cassette were used to check homologous recombination (Supplementary Materials Table S1). The primer pair (CKniaDin/CKniaDout) with the genomic DNA from the wild-type B05.10 isolate was used as a positive control. As a negative control, the primer pair (CKniaDhph/CKniaDout) with the genomic DNA from the wild-type B05.10 isolate was used (Supplementary Materials Figure S1B). A Thermo Scientific O’GeneRuler Express DNA Ladder (ref.SM1563, Thermo Scientific, Waltham, MA, USA) served as the molecular weight marker (Mw). Results are represented as the total number of homokaryotic and heterokaryotic colonies for each of the *B. cinerea* isolates studied, taking five independent transformations into consideration.

### 4.8. Statistical Analysis

Statistical analyses were conducted using GraphPad Prism 8. Normality was assessed using the Kolmogorov-Smirnov test for samples larger than 50 and the Shapiro-Wilk test for samples smaller than 50. Based on the normality results, either the T-test or Mann-Whitney test was employed for comparing normally distributed or nonparametric data, respectively. A significance threshold of *p* < 0.05 was utilized to determine statistical significance.

## 5. Conclusions

This article describes the optimization of the method used for protoplast isolation in non-sporulating isolates B459 and B371. Two days of incubation of five mycelium plugs in Petri dishes containing 20 mL of medium and orbital shaking at 40 rpm was determined to be the best culture method for protoplast isolation. 0.2 g of VinoTaste Pro enzyme in 20 mL of KC buffer for 2 g of mycelium was likewise determined to be the best substitute for the classic Glucanex enzyme used in *B. cinerea*. In this paper, we describe a method capable of generating viable protoplasts in non-sporulating isolates that is useful in applications such as CRISPR/Cas9-mediated transformation.

## Figures and Tables

**Table 1:** Efficiency of the transformations performed on isolates B05.10, B459 and B371.

Isolate	Condition	False Positive	No. of Colonies with *hph* Insertion	No. of Heterokaryon Colonies	No. of Homokaryon Colonies	Total Number of Colonies Screened	Transformation Efficiency (Percentage of Mutants)	Transformation Efficiency (Percentage of Homokaryon)
B05.10	No DNA	12	0	-	-	12	0%	-
	pNDH-OGG	13	2	-	-	15	13%	-
	No DNA + Cas9	10	0	-	-	10	0%	-
	pNDH-OGG + Cas9	21	4	-	-	25	16%	-
	No DNA + Cas9 + *BcniaD* sgRNA	10	0	-	-	10	0%	-
	pNDH-OGG + Cas9 + *BcniaD* sgRNA	2	49	40	9	51	96%	17%
B371	No DNA	12	0	-	-	12	0%	0%
	pNDH-OGG	15	1	-	-	16	6.3%	-
	No DNA + Cas9	10	0	-	-	10	0%	-
	pNDH-OGG + Cas9	20	3	-	-	23	13.3%	-
	No DNA + Cas9 + *BcniaD* sgRNA	10	0	-	-	10	0%	
	pNDH-OGG + Cas9 + *BcniaD* sgRNA	1	51	44	7	52	98%	13.5%
B459	No DNA	13	0	-	-	13	0%	0%
	pNDH-OGG	14	2	-	-	16	12.5%	-
	No DNA + Cas9	10	0	-	-	10	0%	-
	pNDH-OGG + Cas9	18	4	-	-	22	18.1%	-
	No DNA + Cas9 + *BcniaD* sgRNA	9	0	-	-	9	0%	-
	pNDH-OGG + Cas9 + *BcniaD* sgRNA	2	52	46	8	54	96.3%	14.8%

## Data Availability

Data are contained within the article and Supplementary Materials.

## Supplementary Materials

The following supporting information can be downloaded at https://www.mdpi.com/article/10.3390/plants13131754/s1, Table S1. List of oligonucleotides used for the transformation experiment. Figure S1. Strategies employed for hygromycin resistance cassette insertion in *B. cinerea* B05.10, B459 and B371 strains. Figure S2. Molecular characterization of *B. cinerea* B459, B371 and B05.10 mutants.
