# Supplementary material for: Improved Protoplast Production Protocol for Fungal Transformations Mediated by CRISPR/Cas9 in Botrytis cinerea Non-Sporulating Isolates
Source: Plants (Basel). 2024 Jun 25;13(13):1754. doi: 10.3390/plants13131754 (PMC11244380; doi:10.3390/plants13131754)
Supplement: Supplementary file 1 [file plants-13-01754-s001.zip › plants-3007744-supplementary.pdf]

Supplementary Table S1. List of oligonucleotides used for the transformation experiment.

Name of the Oligo	Sequence	Description	References
sgRNAConstantOligo	AAAAGCACCGACTCGGTGCCACTTTTTCAAGTTGATAACGGACTAGCCTTATTTTAACTTGCTATTTCTAGCTCTAAAAC	Oligo used for the synthesis of all the sgRNA. Binds cas9 to the oligo for site-specific recognition of the cleavage site.	[1]
sgRNA1niaD	AAGCTAATACGACTCACTATAGGAAGCGTGAAATCTCCATCGGTTTTAGAGCTAGAAATAGCAAG	Oligo used for guide RNA synthesis to make the cut at the *niaD* locus.	[2]
sgRNA2niaD	AAGCTAATACGACTCACTATAGGCCAGTCAGCCGAATAAGTCGGTTTTAGAGCTAGAAATAGCAAG	Oligo used for guide RNA synthesis to make the cut at the *niaD* locus.	[2]
sgRNA3niaD	AAGCTAATACGACTCACTATAGGAGGAAAACGATATTCGACGAGTTTTAGAGCTAGAAATAGCAAG	Oligo used for guide RNA synthesis to make the cut at the *niaD* locus.	[2]
CKhphpndhFw	GGTAAATAGCTGCGCCGAT	Oligo used to amplify a fragment of the *hph* (hygromycin resistant) gene to check the random integration of foreign DNA	This work
CKhphpndhRv	TGTGCACGGCGGGAGATG	Oligo used to amplify a fragment of the *hph* (hygromycin resistant) gene to check the random integration of foreign DNA	This work
CKniaDout	TTGGTTTCGGCAGATAGATACAG	Oligo outside the recombination region at the *niaD* locus to check the presence of the mutation or wt copies.	This work
CKniaDin	GATATTGTCGGATTTGGGATGG	Oligo inside the recombination region at the *niaD* locus to check the presence of the wt copies.	This work
CKniaDhph	GGTAAATAGCTGCGCCGAT	Oligo inside the recombination region at the *niaD* locus to check the presence of the mutation (correct integration of the *hph* gene at the *niaD* locus).	This work

Supplementary Figure S1. Strategies employed for hygromycin resistance cassette insertion in *B. cinerea* B05.10, B459 and B371 strains. (**A**) Representation of the pNDH-OGG plasmid as exogenous DNA used for CrisprCas9 transformations. In purple letters, primers (CKhphpndhFw/ CKhphpndhRv – amplification of 141 bp) used for detection of the hygromycin resistance cassette in the random insertion transformation strategies as well as damage repair are represented. (**B**) Strategy used for targeted incorporation of the hygromycin resistance cassette together with GFP flanked by the OliC promoter and the T-glu terminator of the pNDH-OGG plasmid. In purple letters are indicated the 3 sgRNAs (sgRNA1niaD-sgRNA3niaD) used in the CrisprCas9 transformation as well as the primer pair for mutation determination (CKniaDhph/CKniaDout – amplification of 1174 bp) and the primer pair for wild-type copy determination (CKniaDin/CKniaDout - amplification of 423 bp).

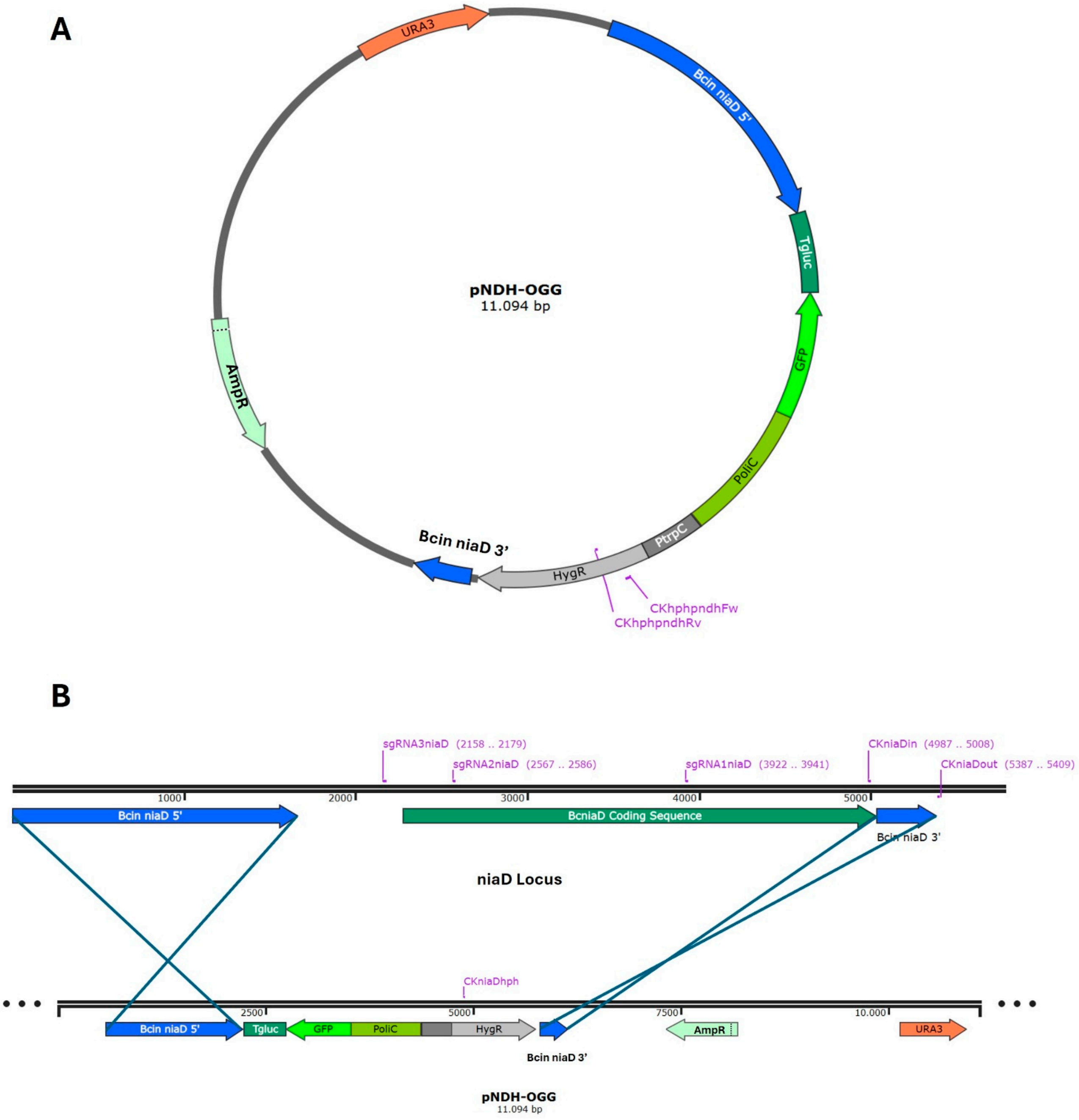

Supplementary Figure S2. Molecular characterization of *B. cinerea* B459, B371 and B05.10 mutants. (**A**) Representation of the amplification of a hygromycin fragment for B05.10, B459 and B371 with the primer pair (CKhphpndhFw/ CKhphpndhRv – amplification of 141 bp). As a positive control the pNDH-OGG plasmid was used as template DNA while the genomic DNA from B05.10 wild-type strain was used as a negative control. The fragments represented correspond to those obtained to verify both in the transformation strategies of random integration of exogenous DNA into the genome and in the (non-directed) DNA repair caused by cas9 cuts. (**B**) Representation of the fragments obtained in the transformation by CrisprCas9, sgRNA-directed. Amplification exclusively with the primer pair (CKniaDhph/CKniaDout – amplification of 1174 bp) indicates the presence of homokaryotic mutants while amplification also with the primer pair (CKniaDin/CKniaDout - amplification of 423 bp), indicates the presence of a wild-type copy, classifying these mutants as heterokaryotic. The primer pair (CKniaDin/CKniaDout - amplification of 423 bp) with the genomic DNA from the wild-type B05.10 isolate was used as a positive control. As a negative control, the primer pair (CKniaDhph/CKniaDout – amplification of 1174 bp) with the genomic DNA from the wild-type B05.10 isolate was used. A Thermo Scientific O'GeneRuler Express DNA Ladder (Thermo Scientific, SM1563) served as the molecular weight marker (Mw).

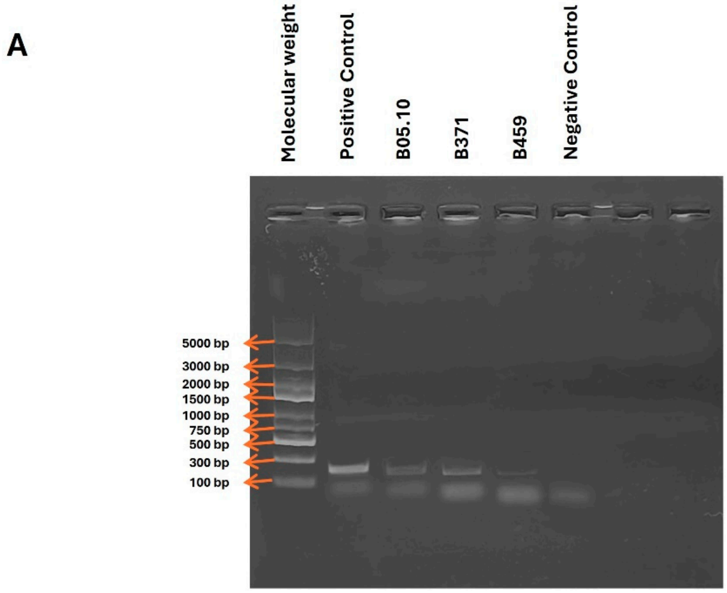

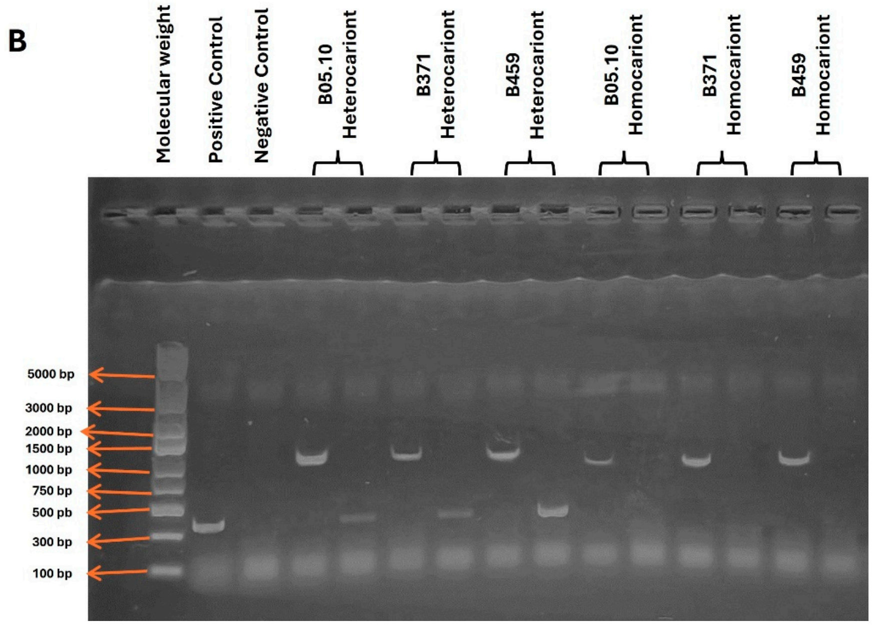

References

1. Savić, N.; Ringnalda, F.; Berk, C.; Bargsten, K.; Hall, J.; Jinek, M.; Schwank, G. *In vitro* generation of CRISPR-Cas9 complexes with covalently bound repair templates for genome editing in mammalian cells. *BIO-PROTOCOL* **2019**, *9*, doi:10.21769/BioProtoc.3136.
2. You, Y. Host resistance mechanisms and fungal infection strategies in the *Botrytis cinerea*-tomato interaction. **2022**, doi:10.18174/570619.
